# Transcriptome datasets from leaves and fruits of the sweet cherry cultivars ‘Bing’, ‘Lapins’ and ‘Rainier’

**DOI:** 10.1016/j.dib.2019.01.044

**Published:** 2019-01-22

**Authors:** Jonathan Maldonado, Amit Dhingra, Basilio Carrasco, Lee Meisel, Herman Silva

**Affiliations:** a Universidad de Chile, Facultad de Ciencias Agronómicas, Departamento de Producción Agrícola, Laboratorio de Genómica Funcional, & Bioinformática, 820808 La Pintana, Santiago, Chile; b Department of Horticulture, Washington State University, Pullman, WA, USA; c Facultad de Agronomía e Ingeniería Forestal, Pontificia Universidad Católica de Chile, Vicuña Mackenna 4860, Casilla 306, Macul, Chile; d Universidad de Chile, Instituto de Nutrición y Tecnología de los Alimentos (INTA), El Líbano 5524, 7830490 Macul, Santiago, Chile

## Abstract

Sweet cherry fruits from different cultivars have different pre- and post-harvest qualities. Here we present the transcriptome profile datasets of leaves and mature fruits of three sweet cherry cultivars (‘Bing’, ‘Lapin’ and ‘Rainier’). Using 454 GS-FLX technology (454 Life Sciences, Roche), transcriptomes of leaves and mature fruits were obtained from these cultivars. These transcriptome data sets are reported here.

**Specifications table**

**Table:** 

Subject area	Biology
More specific subject area	Plant biology; Bioinformatics; Agronomy
Type of data	Table, text file, graph, figure
How data was acquired	RNA deep sequencing, 454 FLX
Data format	Raw
Experimental factors	Extraction of total RNA from leaves and fruit of Bing, Lapins and Rainier sweet cherry cultivars.
Experimental features	Shotgun sequencing of total RNA followed by bioinformatics analysis for differential gene expression
Data source location	La Palma Experimental Station, Faculty of Agronomy PUCV, Quillota (latitude 32º 54׳ S and longitude 71º 12׳ W); Los Andes (latitude 32° 49׳ S and longitude 70° 35׳ W) and San Francisco de Mostazal (latitude 33° 59׳ S and longitude 70° 41׳ W).
Data accessibility	The nucleotide sequences of raw reads were submitted to NCBI’s Sequence Read Archive through the BioProject PRJNA497458 (https://www.ncbi.nlm.nih.gov/bioproject/?term=PRJNA497458) and RNAseq data were submitted to NCBI’s Gene Expression Omnibus database with the accession number GSE121997 (https://www.ncbi.nlm.nih.gov/geo/query/acc.cgi?acc=GSE121997).
Related research article	J.C. Rios, F. Robledo, L. Schreiber, V. Zeisler, E. Lang, B. Carrasco, H. Silva, Association between the concentration of n-alkanes and tolerance to cracking in commercial varieties of sweet cherry fruits, Scientia Horticulturae, 197 (2015) 57-65. http://dx.doi.org/10.1016/j.scienta.2015.10.037

**Value of the data**

• These sweet cherry transcriptome datasets may be used to improve the genome assembly and annotation of *Prunus avium* [1].
• Transcriptome datasets may be used to mine for Single Nucleotide Polymorphisms (SNPs) that are polymorphic between these sweet cherry cultivars.
• Transcriptome datasets may be used to identify differentially spliced transcripts in sweet cherry leaves and fruits.
• These datasets may be used to identify differentially expressed genes that may correlate with the phenotypic variabilities among these and other sweet cherry cultivars.
• Profiling of leaf and fruit transcriptome datasets may be used to identify genetic targets for molecular markers assisted breeding programs [2], [3], [4].

## 1 Data

Here we report the transcriptome datasets of leaves and fruits of three sweet cherry cultivars (‘Bing’, ‘Lapin’ and ‘Rainier’) with contrasting pre- and post-harvest fruit qualities (Table S1). Total RNA sequencing resulted in 956,609 total raw reads, comprising 362 Mb of data, with an average read length of 378 bp. After trimming the number of reads was 938,279 (98% of raw reads) and the average read length was found to be 380 bp. Reads mapped against *Prunus avium* genome predicted genes (n = 43,673 genes) [1] correspond to 55% of filtered reads and covered on average 24% of all genes in all samples. The summary of the data is listed in Table 1. Predicted genes were also functionally annotated using Blast2GO (Table S2).

**Table 1:** Statistics of the sweet cherry transcriptome datasets.

Feature	Leaf			Fruit		
Cultivar	‘Bing’	‘Lapins’	‘Rainier’	‘Bing’	‘Lapins’	Rainier
NCBI Bioproject ID	PRJNA497458					
NCBI Biosample ID	SAMN	SAMN	SAMN	SAMN	SAMN	SAMN
	10258413	10258416	10258418	10258415	10258417	10258419
NCBI SRA ID	SRX4958445	SRX4958446	SRX4958447	SRX4958448	SRX4958449	SRX4958450
NCBI GEO ID	GSE121997					
Sequence type	454 FLX (454 Life Sciences, Roche)					
Total number of reads (raw)	165,657	143,076	143,988	208,854	144,893	50,141
Read length (raw) in base pairs	383.3	361.4	378.0	411.3	369.9	365.9
Total number of reads (filtered)	162,773	140,205	141,408	205,440	141,677	146,776
Read length (filtered) in base pairs	384.0	362.7	378.5	412.5	371.8	367.5
Total number of mapped reads	90,761	71,694	71,706	118,019	75,862	85,481
% of mapped reads	55.8%	51.1%	50.7%	57.4%	53.5%	58.2%
Number of genes with mapped reads	11,231	10,365	10,117	11,086	9276	9600
% of genes with mapped reads	25.7%	23.7%	23.2%	25.4%	21.2%	22.0%

**Fig. 1:**
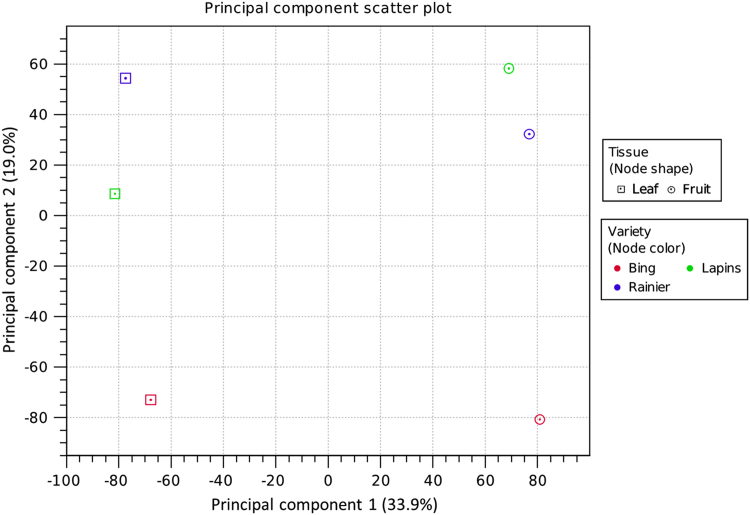
Principal component scatter plot comparing the predicted genes in the transcriptome datasets from leaf and tissue samples of ‘Bing’, ‘Lapins’ and ‘Rainier’ sweet cherry cultivars.

A Principal Component Analysis of the predicted genes from all the tissue samples in the sweet cherry cultivar transcriptome datasets was performed in order to determine the similarities between the whole RNA profiles of these samples (Fig. 1).

We analyzed the Differentially Expressed Genes (DEGs) of these transcriptome datasets using three criteria for their expression profiles: (i) the number of reads that maps to each contig was more than or equal to 10 when compared to another cultivar (dual comparison); (ii) the average fold change in transcript accumulation was more than or equal to 2-fold between cultivars and (iii) the Z-Test p-value was less than or equal to 0.05. Under these criteria, 138 and 52 genes were identified to be differentially represented in the pairwise comparison of the leaf or fruit transcriptome datasets, respectively (Table 2 and Table 3). The transcriptomic analyses for all samples are available in Table S3 and Table S4. Table S5 and Table S6 contain the complete list of genes that are differentially represented in these transcriptome datasets using the pair-wise comparison of the cultivar specific transcriptome datasets of fruit and leaf tissues, respectively.

**Table 2:** Number of genes differentially represented in the pairwise comparison of the fruit transcriptome datasets of three sweet cherry cultivars. The cutoff was: fold of change ≥ 2X and p-value < 0.05.

	**Number of over-represent genes respect to**			
**FRUIT**	**‘Rainier’**	**‘Rainier’ and ‘Bing’**	**‘Bing’**	**Total**
**‘Lapins’**	219	11	11	241
	**‘Lapins’**	**‘Lapins’ and ‘Rainier’**	**‘Rainier’**	
**‘Bing’**	64	65	55	184
	**‘Bing’**	**‘Bing’ and ‘Lapins’**	**‘Lapins’**	
				
**‘Rainier’**	168	5	2	175

**Table 3:** Number of genes with differentially represented in the pairwise comparison of the leaf transcriptome datasets of three sweet cherry cultivars. The cutoff was: fold of change ≥ 2X and p-value < 0.05.

	**Number of over-represented genes respect to**			
**LEAF**	**‘Rainier’**	**‘Rainier’ and ‘Bing’**	**‘Bing’**	**Total**
**‘Lapins’**	90	6	3	99
	**‘Lapins’**	**‘Lapins’ and ‘Rainier’**	**‘Rainier’**	
**‘Bing’**	31	19	10	60
	**‘Bing’**	**‘Bing’ and ‘Lapins’**	**‘Lapins’**	
				
**‘Rainier’**	39	9	10	58

## 2 Experimental design, materials, and methods

### 2.1 Plant material

Sweet cherry leaf and fruit samples from the ‘Bing’ cultivar were collected from La Palma Experimental Station, Faculty of Agronomy PUCV, Quillota (latitude 32º 54׳ S and longitude 71º12׳ W). Samples from the ‘Lapins’ cultivar were collected from Los Andes (latitude 32°49׳ S and longitude 70°35׳ W). Samples from the ‘Rainier’ cultivar were collected from San Francisco de Mostazal (latitude 33°59׳ S and longitude 70°41׳ W). The fruits from these three cultivars were harvested at commercial maturity. All the fresh samples were frozen in liquid nitrogen and stored at -80°C until used for total RNA isolation. These cultivars were selected because they are commercially important cultivars that have contrasting pre- and post-harvest fruit quality (Table S1).

### 2.2 Library construction and deep sequencing

The total RNA was isolated using the protocol of Meisel et al. [5]. The quality and quantity of RNA was determined spectrophotometrically (A260/A280 = 1.8 and A260/A230 = 2.0) and electrophoretically using denaturing formaldehyde agarose gel.

Library construction and 454 FLX deep sequencing (454 Life Sciences, Roche) was performed by the Center for Integrated Biotechnology, Washington State University, using 1/8 plate. Trimming and quality filters were applied to the sequences using the CLC Genome Workbench software, version 11.0.1 (CLC Bio [http://www.clcbio.com]) [6].

### 2.3 RNA sequence analysis

The predicted coding sequences within the *Prunus avium* genome (43,673 predicted genes [1]) were used as reference sets to map the transcripts of these transcriptome datasets. The sequences from each cultivar and tissue were separately mapped against the corresponding reference transcriptome using RNA-seq function of the CLC Genome Workbench version 11.0.1 under the following parameters, similarity: 0.9; length fraction: 0.6; insertion/deletion cost: 3; mismatch cost: 3 and unspecific match limit: 10.

The relative transcript abundance in these datasets were obtained as the unique number of reads mapped to each gene. The transcript abundance in these datasets were compared using a Z-Test [7]. This test compared read counts by considering the proportions in which they make up the total sum of counts in each dataset, correcting for the size of the dataset. For visual inspection, the relative transcript abundance values were transformed using the Log10 method and then normalized by the Quantile method that was the best to fit the result [8], [9].

### 2.4 Functional annotation

Functional annotation was performed on the genes differentially represented in the pairwise comparison of the leaf and fruit transcriptome datasets, using the coding regions predicted in the *Prunus avium* genome [1] and the Blast2GO CLC plugin version 1.11.9 [10] as described in [11]. A best-hit annotation was determined for the differentially represented gene sets by using these genes in a BLASTX (version 2.6.0) analysis against the nr NCBI database with an e-value cutoff of 1e^-6^. INTERPROSCAN analysis (version 5.31-70) with Blast2GO default parameters were also performed [12]. Blast2GO was also used for gene ontology mapping, with the program defaults being applied for all annotation steps and a False Discovery Rate (FDR) cut-off at the 0.05% probability level. The data from the INTERPRO terms, enzyme classification codes (EC), and metabolic pathways (KEGG, Kyoto Encyclopedia of Genes and Genomes) were merged with GO terms to provide a larger accumulation of evidence to support the annotations represented in the supplemental tables (Table S2).

## Appendix B Supplementary materials

The following KMZ files contain the Google maps of the most important areas described in this article.

Map KMZ file containing the Google map.
