# Supplementary material for: Transcriptome datasets from leaves and fruits of the sweet cherry cultivars ‘Bing’, ‘Lapins’ and ‘Rainier’
Source: Data Brief. 2019 Jan 22;23:103696. doi: 10.1016/j.dib.2019.01.044 (PMC6369327; doi:10.1016/j.dib.2019.01.044)
Supplement: Supplementary file 1 — Supplementary material [file mmc1.docx]

**Submission declaration and verification**

The work described in this manuscript has not been published previously (except in the form of an abstract, a published lecture or academic thesis) that it is not under consideration for publication elsewhere, that its publication is approved by all authors and tacitly or explicitly by the responsible authorities where the work was carried out, and that, if accepted, it will not be published elsewhere in the same form, in English or in any other language, including electronically without the written consent of the copyright-holder.
